# Evaluation of the contemporary cultural landscape based on multi-dimensional value coupling: Case study on the Grand Canal, Hangzhou section

**DOI:** 10.1371/journal.pone.0330248

**Published:** 2025-08-18

**Authors:** Wenli Dong, Wenying Han, Yue Xu, Jiwu Wang

**Affiliations:** 1 Department of Regional and Urban Planning, Zhejiang University, Hangzhou, Zhejiang, China; 2 Hangzhou Canal Comprehensive Conservation, Development and Construction Group Co., Shangcheng District, Hangzhou, Zhejiang, China; Zhejiang A and F University, CHINA

## Abstract

In the context of building the Grand Canal National Cultural Park, the heritage conservation of the Grand Canal has entered a new stage since its inscription as a World Heritage site. Scientifically evaluating the value of various cultural landscape projects along the Grand Canal is the fundamental basis for coordinating the conservation of the Grand Canal’s World Heritage sites, land use control, and urban development construction. Based on the research on the evolution of cultural landscape value paradigm, the multi-dimensional value evaluation system of the Grand Canal cultural landscape is constructed. Based on the coupling coordination degree theory, the synergistic adjustment mechanism of the conservative value and developmental value system of the canal cultural landscape is explored. The value evaluation of the 10 contemporary cultural landscape projects implemented since the application of the Grand Canal in Hangzhou is carried out, and the coupling coordination of their conservation and utilization is analyzed. The cultural landscape conservation and utilization mode of the Grand Canal in Hangzhou is discussed, which provides a reference for the cultural landscape construction along the “city-river interdependence” Grand Canal.

## Introduction

In 2014, Beijing-Hangzhou Grand Canal was successfully inscribed on the World Heritage List. In 2019, the State Council of the People’s Republic of China issued a directive aiming to establish the Grand Canal National Cultural Park by the end of 2023. The conservation of the Grand Canal heritage has entered a new phase in the post-bidding era [1]. In response to contemporary development needs, Hangzhou’s urbanization and the “People’s Canal” initiative necessitate further urban renewal and cultural landscape enhancements along the Canal. With the successive promulgation of documents such as the “General Rules for Land Space Control in the Core Monitoring Zone of the Grand Canal in Zhejiang Province” and the “Rules for Land Space Control in the Core Monitoring Zone of the Hangzhou Section of the Grand Canal,” the issue of value conflict between heritage preservation of the Grand Canal and the pressures of local regional development has become increasingly prominent. (1) Due to the special characteristics of Hangzhou’s “city-river dependence”, the boundary effect of the river is much larger than its center effect, and the function of the space along the Grand Canal is separated from that of the urban area. Hangzhou, as the end point of the Grand Canal, where the Grand Canal passes through the densely built-up areas of the main city. This is quite different from that of other cities where the river predominantly runs through the countryside. Since the Tang and Song dynasties, the canal has served as a transportation channel for people and logistics across the region. Therefore, the urban development of Hangzhou relies on the canal, with a high degree of unity between the city and the river. In modern society, the transportation function of the canal has gradually been replaced by the road transportation. The interaction between the canal and the urban development has gradually weakened, which give rises to the spatial fragmentation of the space along the river with that of the urban area.(2) In the context of spatial control of the national territory in China, the development of the areas around the Canal and the Northern City Area has been greatly constrained. The relevant plans issued one after another, such as the General Regulations and the Detailed Rules, which have made the control of urban space along the Grand Canal more stringent and refined. On the other hand, the huge belt-shaped core monitoring zone set up along the river poses a greater challenge to the construction and development of the urban space along the canal. The increasing risk of the boundary effect is further increased. Based on the city-river interdependence of the Grand Canal, the development of its neighboring districts and even the northern part of the city has been greatly constrained. In this background, flexible adjustment between development requirements and control requirements, could systematically improve the construction of the landscape along the river. In summary, the optimization of the conflict adjustment mechanism is supposed to effectively balance the conflicts between the conservation, utilization and development of the cultural landscape of the Grand Canal. In this background, the challenge lies in making informed judgments on various landscape projects along the canal and leveraging cultural landscape initiatives to foster canal-side development while safeguarding canal-related cultural resources and nostalgic canal memories. This issue stands as a pivotal concern in the planning and development of the Hangzhou section of the Grand Canal today.

The landscape of the Grand Canal represents a cultural landscape that integrates natural environments with historical and cultural significance. American geographer Carl Sauer first defined cultural landscape in cultural geography as “the form of human activities superimposed on the natural landscape, a complex created over a specific period, reflecting regional characteristics, and shaped by natural and human factors” [2]. In China, Li Xudan, a pioneer in modern human geography, expands on this definition, viewing cultural landscape as a complex of cultural phenomena on Earth’s surface that reveals a region’s geographic traits, essential for analyzing human-land relationships [3]. This perspective underscores the importance of cultural landscape in understanding the interactions between human activities and their environments. In essence, the cultural landscape of the Grand Canal embodies a synthesis of natural elements, historical evolution, and human cultural practices over time. It stands as a collective achievement where human ingenuity harmonizes with natural surroundings, thereby possessing inherent value.

The study aims to develop a comprehensive cultural landscape value evaluation system that integrates multiple dimensions of value coupling between conservation and utilization. This system will be applied to assess ten cultural landscape projects implemented in Hangzhou since the bidding for Grand Canal heritage, under the unique conditions of Hangzhou’s “city and river dependence.” The goal is to investigate value evaluation and adaptation mechanism, conservation and utilization synergy mechanism, coordination and management mechanism for the cultural landscape of the canal, so that the conservation, inheritance and utilization of the cultural landscape are compatible with each other.

**Fig 1:**
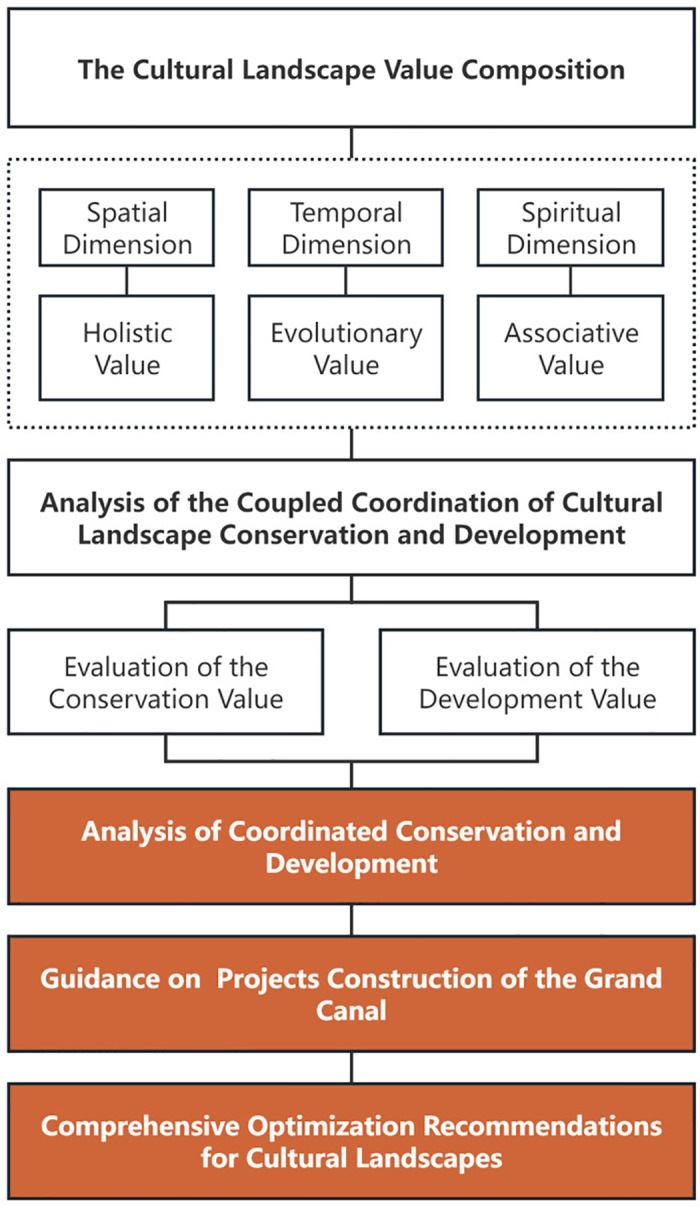
Logical framework of the research.

The study starts from the cultural landscape paradigm research and value composition analysis, establishes a framework for the analysis of cultural landscape conservation and development coupling & coordination, selects the value index system based on quantitative and qualitative grading, and carry out coupling & coordination analysis; based on the comprehensive results, guidance and suggestions for the key projects of the canal are put forward to holistically optimize the cultural landscape of the canal (Fig 1).

## Literature review: A cognitive study of multidimensional value evaluation in cultural landscapes

### Philosophical perception of the value theory

Philosophical understanding of value has evolved from focusing on inherent attributes and functions of objects to emphasizing subjective perspectives and life practices [4]. Traditional research on the value of cultural landscapes has historically aligned with philosophical value theory. Over time, this research has expanded into multidimensional dimensions with the introduction of various conservation frameworks.

Initially centered on historical, artistic, and archaeological intrinsic values, the evaluation of cultural landscapes has progressively broadened into social and cultural contexts [5]. This recognition manifests across spatial, temporal, and spiritual dimensions [6,7]. Spiritual value, encapsulated by the concept of locality, underscores the cultural landscape’s role as a material repository imbued with history, place, and identity [7]. The study of contemporary landscapes is particularly relevant to the value of time.

In terms of temporal value, the theory of Historic Urban Landscape (HUL) integrates the temporal dimension of landscapes into the value dimension of cultural landscape spaces, forming a dual-dimensional framework for analyzing the temporal and spatial values of cultural landscapes. The approach underscores the dynamic interaction between people and landscapes, driving cultural landscape evolution. The spatio-temporal heritage model integrates geographical knowledge and landscape heritage, introducing ephemerality and co-temporality in evaluating historical and spatial dimensions [8,9]. Cultural landscapes are rich in historical value because they accumulate values and attributes during the process of historical development [8]. Nowadays however, the participation of human activities still drives the development of cultural landscapes from time to time, so their time-evolving value is not a simple superposition of historical elements, but embodies certain usability and development. The Grand Canal is a living, in-use cultural corridor. With the development of the city, a large number of modern buildings along the Grand Canal have become new landmarks in the city. Together with the historical and cultural resources such as historical districts, cultural heritage units and historical buildings along the canal, which have a wide recognition and spiritual symbolism, they have become the anchors of the cultural landscape along the Grand Canal in the contemporary times.

The study of spatial value is the main focus. It primarily focuses on dimensions such as natural space, built space, and interactive human-environment space. It analyzes the inherent natural value, landscape value, and other aspects within cultural landscape spaces, and integrates diverse theories such as spatial syntax, spatial composition studies, and semiotics. Methodologically, the evaluation in spatial dimensions is based on Landscape Character Assessment (LCA) [10]. Landscape character is the recognizable, unique, and consistent elements of a landscape. It is not used to describe the superiority or inferiority of two landscapes, but rather to emphasize the difference between one landscape and another. Related methods also include the Historic Landscape Character Recognition System (HLC) and the Historic Townscape Character Assessment System (HTC), etc. Conzeen (1966) established a three dimensional system including units, building types, and land use types [11].

### Definition on the value of cultural landscapes

**Fig 2:**
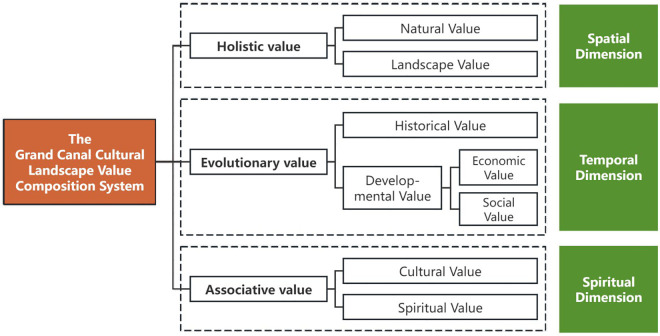
Value composition system of cultural landscape along the Grand Canal.

The value of cultural landscapes derives from their combination of humanistic and natural elements, influenced by the interaction of time, space, and spirit. Through a review of literature on cultural landscape evaluation and traditional Chinese landscape characteristics, the value system of canal cultural landscapes is identified. This system includes holistic value in the spatial dimension, evolutionary value in the temporal dimension, and associative value in the spiritual dimension. The holistic value includes natural and landscape aspects, corresponding to the nature and artificiality; the evolutionary value includes historical and developmental dimension, corresponding to the history and the future; and the cognitive value includes cultural and spiritual areas, corresponding to the traditions and beliefs (Fig 2).

Take spiritual value for example, it contains spiritual value of the place, folk belief value, artistic spiritual Value, and national self-confidence. Spiritual value of the place indicates the general awareness and spiritual sense of belonging of the public to the place of the landscape space. Folk belief value reflects the richness of folk beliefs contained in the cultural landscape. Artistic spiritual value refers to imagery spiritual value that contained in poetry, song, fugue, painting and other related art works.

### Cultural landscape value assessment and coupling

The theory of multidimensional value-based conservation planning restructures the traditional linear process of heritage conservation by centering it around “values” [12]. With the emergence of new forms of cultural heritage, such as cultural landscapes, and the increasing complexity of their associated interests and conflicts, there is a pressing need to incorporate theoretical tools that facilitate the harmonization of these values.

Coupling coordination degree is employed to gauge the level of coordination among interrelated entities that mutually influence each other. A higher coupling coordination degree indicates greater balance and health between these entities, whereas a lower degree signifies the opposite. Maintaining system coordination is crucial for its health and sustainable development [13]. Building upon the theory of coupled coordination, research has explored the interrelations between cultural heritage and the tourism industry [14], the value of cultural heritage and tourism [15], the intersection of tourism industry and new urbanization [16]. Through analysis of coupling and coordination degrees, these studies aim to assess the level of coordinated development between these dual systems and inform path selection.

**Fig 3:**
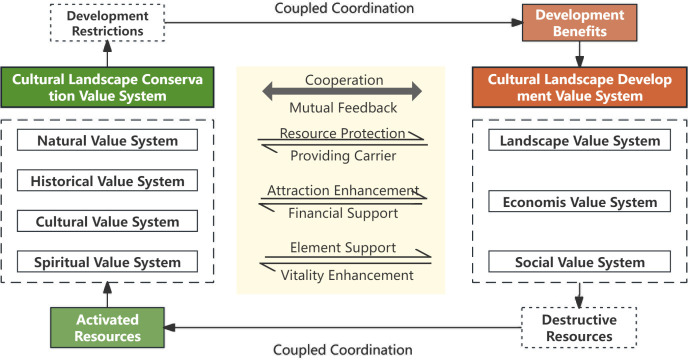
Value system framework of the cultural landscape along the Grand Canal based on the theory of conservation-development coupling & coordination.

To address the conflict between conservation and utilization in cultural landscape practices, this study focuses on the “conservation-development” dual value system of cultural landscapes. The value systems of conservation and development in cultural landscapes are inherently complex and often contradictory [17]. When effectively coordinated, these dual systems can synergistically support each other: the conservation system provides resources, enhances gravitational pull, and supports foundational elements for development, while the development system offers spatial platforms, financial backing, and injects vitality into the conservative efforts. Simultaneously, these dual systems of conservation and development impose constraints on each other. The conservative system regulates landscape development through element conservation and spatial controls, while the developmental system impacts elemental resources during landscape construction and economic development. Leveraging the theory of coupling coordination degree, this study aims to define, measure, adapt, and harmonize the conservation and development value systems of cultural landscapes. This approach seeks to optimize the interaction between these systems, balancing gains and losses (Fig 3).

Coupling Coordination Degree Model (CCDM) is used to measure the degree of interaction between two or more systems. In Coupling Coordination Degree Model (CCDM), the coupling-coordination level is usually classified based on the D-value (Coupling Coordination Degree, range [0,1]) to quantify the level of synergistic development between systems. However, the traditional coupling-coordination degree division may have the problems of relying on empirical division thresholds, uneven data distribution, and inconsistent standards, which lead to subjective and unreasonable division of intervals, and difficult to compare horizontally. Uniform Distribution Function Method (UDFM) is an analytical method based on probability statistics, which is mainly used to describe the random variables that have equal probability of taking all values in a certain interval. Uniform distribution is often used to simulate random experiments (such as random sampling, Monte Carlo simulation, etc.), to ensure that the data in a given range of uniform distribution, to avoid bias. The uniform distribution function method can be automatically divided based on the probability distribution, reducing human intervention. The interval can be adjusted according to the actual distribution of the data, improving the scientific nature of the classification. It is applicable to different research cases, enhancing the comparability of the results. Thus, it can provide an objective, standardized, adaptable, and divisible way for the analysis of coupling-coordination degree. The threshold of coupling-coordination level needs to be flexibly defined by combining the characteristics of the domain and the data distribution. The uniform distribution method can enhance the objectivity, the rationality of the division, however, still needs to be verified by empirical evidence in the actual research. For example, the K-S test (Kolmogorov-Smirnov Test) could be used to ensure that the data are uniformly distributed within the interval. The sensitivity analysis could adjust the threshold value to observe the stability of the results.

### Evaluation on conservation and development value for cultural landscape projects of the canal

#### Establishing the evaluation framework.

Integrating literature review and field research, this study adheres to principles of typicality, systematicity, scientific rigor, and practicality, while taking into account the principle of differentiation oriented to the territorialization of landscapes, uncertainty of value assessment. The selected evaluation indicators reflect the multiple dimensions of the cultural landscape in terms of time, space and spirit, which represents the “systematic” nature of the indicators. On the one hand, the typicality is reflected in the fact that most of the indicators are derived from regulations and planning documents related to the Grand Canal, and optimized by expert questionnaires, which ensures the comprehensiveness of the system as well as the simplicity and representativeness of the indicator system. The scientificity is reflected in the optimization process of the indicator system by adopting the expert questionnaire. Practicality is mainly reflected in the availability of indicator data and the convenience for calculation.

It selects evaluation indices that encompass both conservation and development values of the cultural landscape. These indices are refined through filtering and consolidation to establish the evaluation system for Hangzhou section (Tables 1–2). Operational Guidelines for the Implementation of the World Heritage Convention, 2021 Edition, Guidelines for the Protection of Cultural Heritage Monuments in China, the Outline of the Plan for the Protection, Inheritance and Utilization of the Culture of the Grand Canal, the Program for the Construction of the Great Wall, the Grand Canal and the Long March National Cultural Parks, and the Plan for the Construction and Protection of the National Cultural Park of the Grand Canal, the “14th Five-Year Plan” Implementation Program, the General Rules for Land Space Control in the Core Monitoring Area of the Grand Canal in each province, as well as other regulations and planning documents related to the Grand Canal, and at the same time, combined with the theoretical research, literature compilation and interviews related to the cultural landscape and the Grand Canal, to initially construct the indicator system of the protective value of Grand Canal cultural landscapes and the developmental value of Grand Canal cultural landscapes.

**Table 1:** Indicators of Conservative Value for Canal Cultural Landscapes.

Type of indicator	Primary indicator	Secondary indicator	Explanation of indicators
**Conservation value**	**Natural value**	**Hydrological value of the river**	Spatial distance of the canal channel affects hydrological value
		**Vegetation index**	Density of vegetation within the cultural landscape area along the canal
		**Ecosystem carbon stocks**	Habitat carbon stocks within the unit
		**River network density**	River network density within the unit
	**Historic Value**	**Historic longevity**	It refers to the age of the cultural landscape, significant historical events and associations with historical figures.
		**Historical rarity**	It refers to the rarity of the cultural landscape heritage type in the watershed
		**Historical status**	It refers to the importance of the cultural landscape in the historical development of the Grand Canal
		**Historical remains**	It refers to the preservation and survival of historical canal buildings and industrial heritage within the cultural landscape.
		**Cultural conservation unit**	Grade and conservation degree of of historical and cultural landscape resource sites, including historical and cultural blocks, national, provincial and municipal cultural conservation units.
		**Canal heritage value**	Classification of the river in the heritage section.
	**Cultural value C3**	**Cultural continuity**	It refers to the culture of the Grand Canal that displayed from the cultural landscape.
		**Cultural diversity**	It refers to the multicultural integration of the cultural landscape during its formation and evolutionary development.
		**Cultural uniqueness**	Canal culture that is unique, scarce and has obvious local characteristics in the cultural landscape.
		**Intangible cultural heritage abundance**	Grade, quantity and density of intangible cultural heritage of the canal
		**Degree of cultural revitalization**	Revitalized display,inheritance and utilization of intangible cultural heritage such as folk art, traditional crafts, traditional activities, etc. along the traditional culture of the Canal.
		**Artistic value**	It refers to the development level of architecture, literature, craftsmanship and other specialized techniques reflected in the cultural landscape.
	**Spiritual value**	**Spiritual value of the place**	The general awareness and spiritual sense of belonging of the public to the place of the landscape space
		**Folk belief value**	It reflects the richness of folk beliefs contained in the cultural landscape.
		**Artistic Spiritual Value**	Imagery spiritual value contained in poetry, song, fugue, painting and other related art works.
		**The value of national self-confidence(D18)**	The positive effect of the “canal spirit” on national self-confidence.

**Table 2:** Indicators of developmental value for canal cultural landscapes.

Type of indicator	Primary indicator	Secondary indicator	Explanation of indicators
**Developmental value**	**Landscape value**	**Integrity of Preservation**	It refers to the degree to which the components and ethos of the cultural landscape are maintained.
		**Diversity of combination**	It refers to the diversity of the cultural landscape itself in terms of structure and function, reflecting the complexity of the combination.
		**Human landscape interaction**	It refers to the degree to which the cultural landscape reflects the harmonious interaction between human beings and nature, and the degree to which human intervention is adapted to nature.
		**Spatial coordination**	Whether the building height and skyline within the cultural landscape meet the height requirements of the Grand Canal zoning district and the requirements of the view corridors of important nodes along the Grand Canal.
		**Landscape coherence**	Whether the architectural style and volume of the cultural landscape are in harmony with the style along the canal and the traditional local architectural style, and whether they meet the aesthetic requirements of the representative interfaces along the Grand Canal.
		**Landscape landmark**	The cultural landscape in the region that embodies the degree of prominent location, outstanding image, strong publicity and rich landmark.
		**Road texture**	Road traffic accessibility
	**Economic value**	**Economic value**	It mainly refers to the economic value that the cultural landscape of the canal can directly generate for the continued use of human beings.
		**Shipping value**	The contribution of the cannal cultural landscape to guarantee and enhance the function of flood prevention and drainage, the optimal allocation of water resources, the conservation of the shoreline and wharf services, etc., so as to improve the navigability of the Canal and the value of regional shipping.
		**Cultural industry-ability**	The degree of innovative and diversified development of cultural industries and cultural exchange platforms that related to the canal.
		**Integration of culture and tourism**	The integration of culture and tourism in the cultural landscape.
		**Development potential**	Sustained economic value brought by further development in the future
	**Social value**	**Educational value**	The spiritual enhancement of the public through visiting and learning from cultural landscapes.
		**Regional value**	Regional Participating and cooperation.
		**Service Value**	Creation of cultural highland and public services provision to improve the quality of life along the canal.

Firstly, evaluation of the Conservation Value is based on its historical and current conditions. It encompasses Natural Value, Historical Value, Cultural Value, and Spiritual Value, which have evolved over long periods of historical development, exhibit stability over extended cycles of change, and hold significant importance for conservation efforts.

Then, based on the developmental conditions of cultural landscape projects, the construction of an evaluation system for cultural landscape value development considers Development Value, which encompasses Landscape Value, Economic Value, Social Value. These values have great potential for change with short change cycles. The system assesses the capacity of cultural landscapes to respond to current and future developmental needs within specific time frames, highlighting the significance of their evolution.

### Indicator screening based on expert consultation scores

**Fig 4:**
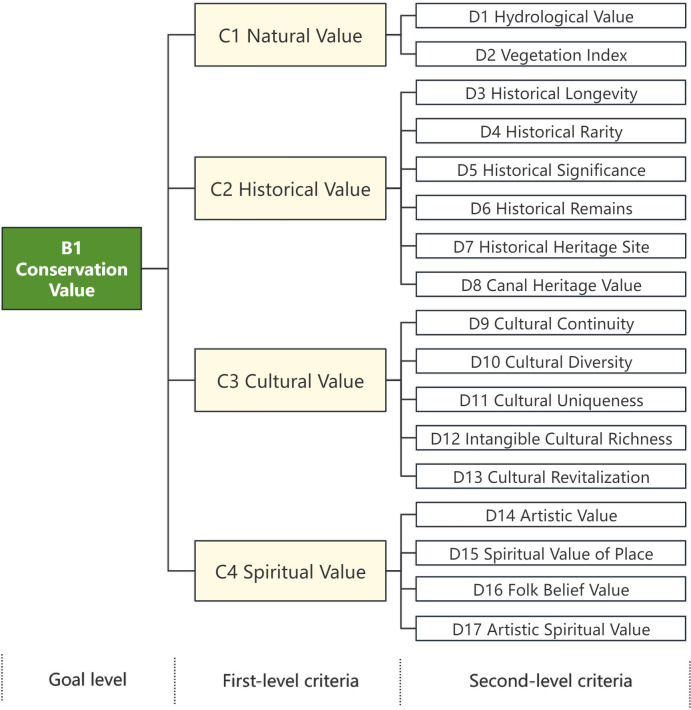
Conservation value system of cultural landscapes of the Grand Canal (Hangzhou section).

**Fig 5:**
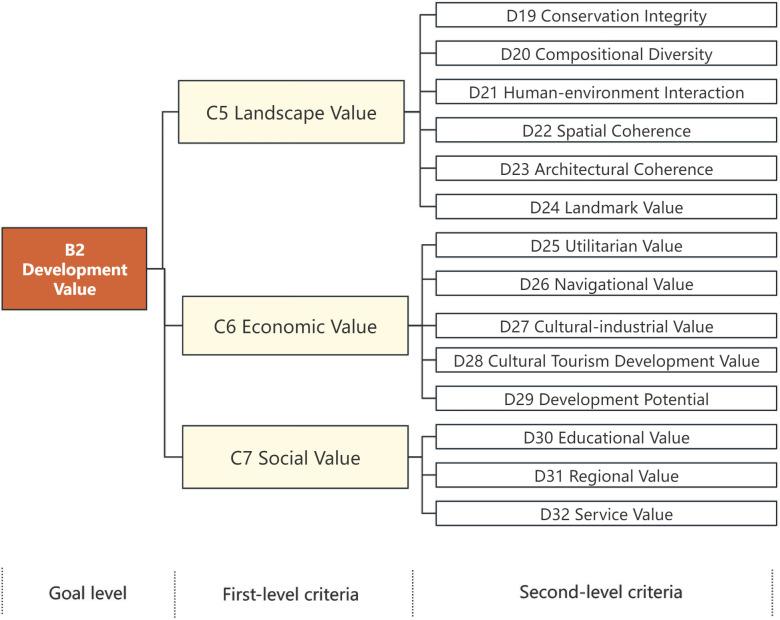
Development value system of cultural landscapes of the Grand Canal (Hangzhou section).

In this paper, experts are invited to express their opinions anonymously through questionnaires in order to carry out the screening and optimization of indicators. Firstly, the initial evaluation index system is formulated based on the literature. Then the expert questionnaire is prepared, and the initial index system is modified after completing the expert consultation. Specifically, 10 experts who have participated in the work related to the Grand Canal are invited to form an expert group. They come from the research departments, design departments and universities, covering urban planning, heritage conservation, civil engineering and architectural design. The questionnaire and indicator descriptions were sent to the experts who were given the opportunity to make decisions on the screening of the indicators. A total of 10 experts in the relevant fields in the expert group were asked to conduct correspondence research. 10 questionnaires were recovered and all of them were valid questionnaires, so the coefficient of the experts’ activeness was 100%.The authority of the experts in the decision-making group was calculated to confirm the scientific validity of the results. The average value of the scores from the invited experts, as well as the coefficient of variation were calculated to screen the indicators. The indicators are ranked according to their degree of importance, from highest to lowest: very important (5 points), relatively important (4 points), generally important (3 points), relatively unimportant (2 points), and very unimportant (1 point). When the average value of the score of the evaluation indicators is less than 3.6 or the coefficient of variation is more than 0.3, it will be excluded. The refining of the indicators will be carried out in combination with experts’ opinions that an indicator needs to be added or deleted. According to the expert questionnaires, the indicators of river network density, and ecosystem carbon stock, the value of road texture, value of national self-confidence is excluded. The refined framework is shown in Figs 4 and 5.

### Determination of weights based on G1 method

The sequential relationship analysis method, generally called the G1 method, is a weight determination method based on sequential relationships, which is designed to improve the consistency problem that may exist in the construction of judgment matrices in the traditional hierarchical analysis method. The relative importance ratio between neighboring indicators is determined by establishing the ordinal relationship of evaluation indicators, thus circumventing the process of constructing complex judgment matrices, simplifying calculations and eliminating the need for consistency testing.

Firstly it requires experts to rank the importance of the indicator set to form a strict chain of ordinal relationships. For the indicator set, the order of importance is determined as Y1 > Y2 > ... > Ym (> means “not inferior”). This process is accomplished by gradually filtering the most or least important indicators to ensure the uniqueness and logical consistency of the ordinal relationship.

After establishing the ordinal relationship, experts need to assign the relative importance ratio R_k_ = Y_k-1_/Y_k_ of neighboring indicators Y_k-1_ to Y_k_. The assignment reference table (Table 3) provides a standardized scale.

**Table 3:** Reference to relative values between indicators.

Rk	Explanation
**1.0**	Indicator Yk-1 is of equal importance compared to Yk
**1.1**	Between equally and marginally important
**1.2**	Indicator Yk-1 is slightly more important than Yk
**1.3**	Between slightly and relatively important
**1.4**	Indicator Yk-1 is more important than Yk
**1.5**	Between more and less important
**1.6**	Indicator Yk-1 is especially important compared to Yk
**1.7**	Between especially important and extremely important
**1.8**	Indicator Yk-1 is extremely important compared to Yk

The weights are calculated by recursive formula:


$$u_{m}={\left(1+\sum_{k=2}^{m}\prod_{i=k}^{m}R_{i}\right)}^{-1},u_{k-1}=R_{k}u_{k}\;\left(k=m,m-1,\ldots ,2\right)$$

The formula is derived backward from the weights of the least significant indicator $u_{m}$ to finally obtain the full weights without constructing a judgment matrix, thus avoiding transmissibility contradictions.

### Data collection and evaluation of indicators

Based on the Conservation Value System of Cultural Landscapes of the Grand Canal (Hangzhou section), this study analyzes various elements that contribute to the cultural landscape’s conservative value. Natural Value Elements assessment considers the average distance from the project to the river and the density of vegetation cover. Historical Value assessment examined the longevity and historical rarity, significance and status, as well as the number of historical remains. This encompasses historical buildings, industrial heritage, Third Heritage Census points, and cultural resource points to be included in the Hangzhou Grand Canal National Cultural Park. Additionally, it assesses the number of cultural preservation units, such as historical and cultural districts, world heritage sites, and cultural preservation sites at national, provincial, and municipal levels. The classification level of river segments within the heritage section is also considered. Cultural Value includes cultural continuity, diversity, and uniqueness, alongside the abundance of non-heritage cultural elements. It also considers the cultural revitalization efforts and the artistic value embedded in the landscape. Spiritual Value assessment covers the spiritual significance of the place, the presence of sacred or faith-based spaces. It also evaluates elements contributing to artistic value, such as poems, songs, paintings, legends, and myths associated with the canal. Finally, it examines how the canal embodies the national spirit.

Based on the Development Value System of Canal Cultural Landscapes, the Developmental elements of the key Cultural Landscapes in Hangzhou Section are analyzed. Landscape Value Elements are evaluated based on their preservation integrity, combination diversity, people-scape interaction, spatial coordination, landscape coordination, and the presence of landscape landmarks. These evaluations are predominantly subjective, combining on-site research and project planning. Economic Value Elements includes factors such as the practical use of the landscape, shipping value, the development of cultural industries, the value it adds to cultural tourism, and its potential for further development. Social Value Elements encompass the landscape’s educational value, their impacts on the surrounding region, and the services they provides to the community.

The evaluation system includes both quantifiable and unquantifiable indicators. Quantifiable indicators encompass measurable attributes such as quantity, density, and age. In contrast, unquantifiable indicators involve characteristics like interactivity, uniqueness, and activation degree. To enhance the evaluation’s practicality, the evaluation employs a five-level categorization ranging from “good” to “bad”. Each indicator is assessed objectively and subjectively, assigning scores from “5” (excellent) to “1” (poor) during the evaluation process. The evaluation criteria are summarized in Tables 1–2. Quantifiable indicators were categorized at 5 levels using the natural breakpoint method. Non-quantifiable indicators utilize weighted statistics based on questionnaire scores from five experts using the expert scoring method. For example, Hydrological value of the river indicates the spatial distance of the canal. Diversity of combination (D20) refers to the diversity of the cultural landscape itself in terms of structure and function, reflecting the complexity of the combination, which is predominantly based on expert questionnaire because of its non-quantizability.

### The coupling and coordination model for the conservation and development values of the cultural landscape

Building upon the value system of the Grand Canal’s cultural landscape, this paper investigates the coupling and coordination model for the conservation and development values of the Hangzhou section.

In this study, the coupling coordination evaluation model contains six quantitative results of the subsystems, namely, the efficacy function f(x), the difference of efficacy indices Z, the normalized efficacy function u(x), the comprehensive evaluation value T, the degree of coupling C and the degree of coupling coordination D.

According to the definition of the coupling degree and coupling coordination degree, D1,D2,...,Dm are m indicators describing the conservative value system of cultural landscape; positive numbers E1,E2,E3,E4,...,En are n indicators describing the developmental value system of cultural landscape.


$$f\left(x\right)=\sum {\mathrm{\backslash nolimits}}_{i=1}^{m}a_{i}x_{i}\;f(y)=\sum {\mathrm{\backslash nolimits}}_{j=1}^{n}b_{j}y_{j}$$

They are the efficacy functions of the conservative value system and the developmental value system, where: ai and bi are respectively the weights of each indicator. The analysis of the efficacy index difference Z based on the conservative value efficacy function f(x) and the developmental value efficacy function f(y), is a crucial value for the categorization of cultural landscape projects. Its function is as follows:


Z=f(x)−f(y)

In the formula, Z is he efficacy index difference; f(x) is the efficacy function of conservative value; f(y) is the efficacy function of developmental value.

Based on the index Z and the value of the efficacy index f(x) and f(y), the cultural landscape projects are categorized into three basic types as conservation, synchronization, and developmental types. Five sub-types are further identified as focused- conservation, conservation-dominant, coordination-synchronization, key development, and development-dominant (Table 4).

**Table 4:** Conservation and Developmental Typology System of Cultural Landscapes along the Canal.

Primary conditions	Basic types	Secondary conditions	Detailed types
f(x)−f(y)>0.5	conservation	f(x)>4	Focused- conservation
		f(x)≤4	conservation-dominant
|f(x)−f(y)|≤0.5	synchronization	–	synchronization
f(x)−f(y)<−0.5	developmental	f(y)>4	key development
		f(y)≤4	development-dominant

In order to make a more objective and detailed analysis on the degree of coupling coordination between the two systems, the uniform distribution function method is used to reclassify the degree of coupling coordination that has been calculated, which is divided into ten levels: extremely out of tune, seriously out of tune, moderately out of tune, mildly out of tune, on the verge of being out of tune, barely coordinated, primary coordinated, intermediate coordinated, well coordinated, and high-quality coordinated. They are respectively categorized into three major strategies: make up for the shortcomings, comprehensive optimization, preservation and use, which guide the construction of the cultural landscape system (Table 5).

**Table 5:** Classification criteria of the coupling- coordination degree of the cultural landscape projects along the Grand Canal.

Coordination degree	Coupling- coordination level	Strategy types
(0−0.1]	Extremely out of tune	Deficiency elimination
(0.1−0.2]	Seriously out of tune	
(0.2−0.3]	Moderately out of tune	
(0.3−0.4]	Mildly out of tune	Comprehensive optimization
(0.4−0.5]	On the verge of being out of tune	
(0.5−0.6]	Barely coordinated	
(0.6−0.7]	Primary coordinated	
(0.7−0.8]	Intermediate coordinated	Preservation and use
(0.8−0.9]	Well-coordinated	
(0.9−1.0]	Quality coordination	

## Case study results: Conservation and development value evaluation of the cultural landscapes projects in Hangzhou section

### Research area

The total length of the Hangzhou section is 261 kilometers, including the World Heritage River, the Grand Canal (Zhejiang section) heritage conservation planning and designing section, as well as part of the state-controlled river, which involves seven urban areas in Hangzhou. As a city where the main canal passes through the old city, Hangzhou and the Grand Canal profoundly embody the characteristic of “city and river inter-dependence”. With the comprehensive conservation and re-development of the landscape along the canal, the Grand Canal has formed an important living heritage conservation corridor and cultural tourism development golden tour route in Hangzhou, which consists of historical heritage sites and modern landscape landmarks.

This study focuses on 10 cultural landscape projects carried out in the Hangzhou section of the Grand Canal since its inscription, including the Centralized landscape boulevard in City North, Hangzhou Steelworks Park, Xiaohe Park, the Grand Canal Waterfront Public Space, Waterborne Industry, the Comprehensive Enhancement for the Old City Section of the Canal, the Beijing-Hangzhou Grand Canal Museum Complex, Canal Bay TOD, Hangzhou Steelworks Station TOD, and the Grand Canal Future Art and Technology Center, which mainly involve the Gongshu District and Shangcheng District. These projects are oriented to the new needs of Hangzhou’s development, targeting the revitalization and utilization of historical heritage space, industrial heritage space and natural space along the river, combining both the ancient and the modern, lines and surfaces. They are typical representatives of the contemporary cultural landscape projects of the Hangzhou section.

The selected cultural landscape projects are mainly those implemented after the year of 2020. According to the control requirements in the Conservation Plan for the World Cultural Heritage of the Grand Canal in Hangzhou, 4 projects belong to the primary buffer zone, 4 belong to the secondary buffer zone, 1 belongs to the environmental control zone (similar to the core monitoring zone and optimized development zone), and there are no projects within the heritage area. According to the Implementation Plan for Cultural Conservation, Inheritance and Utilization of the Grand Canal in Hangzhou, 4 projects are located in the buffer zone; 5 projects are located in the core monitoring zone; and 1 project belongs to the optimized development zone. According to the “Rules for Land Space Control in the Core Monitoring Zone of the Grand Canal of Hangzhou”, 10 projects are in the city built-up areas in the core monitoring zone, 9 of which are historical and cultural conservation zones, and 6 of which are also in the cultivation zones of new canal functions. The other one is located in the general urban built-up area. Therefore, overall the 10 projects are mainly in the buffer zone and core monitoring area. There are no projects within the heritage area. In addition, the Grand Canal Hangang Park is included in the analyzed cases because it is located in the New Function Cultivation Zone in the “Grand Canal Core Monitoring Area” (Table 6). Based on the Grand Canal Cultural Landscape Value Indicator System constructed in Section 3, the relevant index data are collected and further processed through ArcGIS and Excel.

**Table 6:** Conceptual analysis of the scope covered by the Protection Plan, Implementation Plan and By-laws.

Heritage Zone	The area is expanded outward from the canal by 5 meters.		
**Buffer Zone**	The heritage area expanded outward from 40 meters to 240 meters.	The first level buffer zone4	Area that is delineated in combination with the current the green line along the river with a focus on green space control and ecological landscape conservation.
		Secondary Buffer Zone	Newly built and renovated sections along the river outside the primary buffer zone
**Core monitoring area**	Built-up urban area	General built-up area of towns1	Territorial space where town development and construction are allowed within a certain period of time
	Special areas of built-up urban areas	Historical and cultural area	Theme display area of the Grand Canal National Cultural Park Control and Protection Zone
		New Function Cultivation Zone	Liangzhu, Wangjiang, Linping, Hangzhou South Railway Station New City: Xiangfu, Xintiandi Sub-center, Binjiang, Xiaoshan Public Service Center
		Harbor transformation area	Linping, Renhe, Chongxian, Ngaqian 4 operational areas
		Riverfront ecological space	Area within 1000 meters of the river except for the built-up areas
	Non-urban built-up area	Area outside the built-up area of the town, within 1–2 km between the start and end lines on both sides of the river.	
**Optimized Development Zone**	Shangcheng District, Xiacheng District, Jianggan District, Gongshu District, Xihu District, Binjiang District, Xiaoshan District Yuhang District		

## Results of value analysis

Using the Conservation-Development Value System of Cultural Landscapes of the Grand Canal (Hangzhou section), this study evaluates the selected 10 cultural landscape projects.

**Fig 6:**
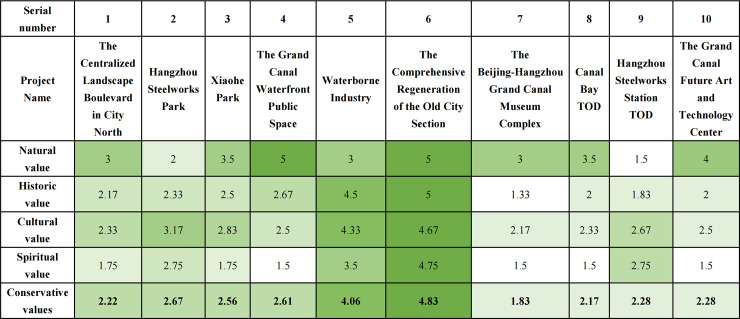
Analysis results of the conservation value for 10 cultural landscape projects of Hangzhou Section.

Conservation Value assessment result reveals varying degrees of conservative value among the projects. The Comprehensive Regeneration of the Old City Section (4.83 points) and Waterborne Industry (4.06 points) demonstrate high conservation value. Hangzhou Steelworks Park (2.67 points), the Grand Canal Waterfront Public Space (2.61 points), and Xiaohe Park (2.56 points) exhibit good conservation value. Conversely, the Beijing-Hangzhou Grand Canal Museum Complex (1.83 points), Canal Bay TOD (2.17 points), the Centralized landscape boulevard in City North (2.22 points), Hangzhou Steelworks Station TOD (2.67 points), and the Grand Canal Future Art and Technology Center (2.28 points) show relatively lower conservation value (Fig 6).

**Fig 7:**
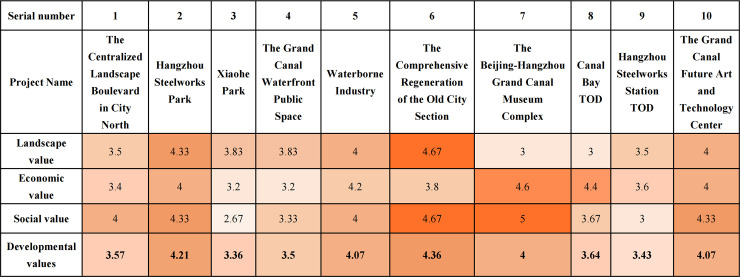
Analysis results of the development value for 10 cultural landscape projects of Hangzhou Section.

The evaluation of Development Value demonstrates relatively good development potential. The Comprehensive Regeneration of the Old City Section (4.36 points), Hangzhou Steelworks Park (3.43points), the Grand Canal Future Art and Technology Center (4.07 points), the Beijing-Hangzhou Grand Canal Museum Complex (4 points), and Waterborne Industry (4.07 points) demonstrate high development value. Projects showing good development value include Canal Bay TOD (3.64 points) and the Centralized landscape boulevard in City North (3.57 points). Conversely, Xiaohe Park (3.36 points), the Grand Canal Waterfront Public Space (3.5 points), and Hangzhou Steelworks Station TOD (3.43 points) are rated as having fair developmental value (Fig 7).

### Research on coupling coordination

This study focuses on analyzing methods for evaluating coupling coordination models. Initially, a coupling coordination evaluation model is selected to assess the interaction between conservation and development aspects within cultural landscape projects. Quantitative results are obtained for each subsystem, including the Coupling Degree C, Coordination Index T, and Coupling-Coordination Degree D (Table 7).

**Table 7:** Analysis of conservation-development coupling coordination degree of 10 cultural landscape projects of Hangzhou Section.

	The Centralized Landscape Boulevard in City North	Hangzhou Steelworks Park	Xiaohe Park	The Grand Canal Waterfront Public Space	Waterborne Industry	The Comprehensive Regeneration of the Old City Section	The Beijing-Hangzhou Grand Canal Museum Complex	Canal Bay TOD	Hangzhou Steelworks Station TOD	The Grand Canal Future Art and Technology Center
**Consolidated average of conservative values**	2.22	2.67	2.56	2.61	4.06	4.83	1.83	2.17	2.28	2.28
**Composite average of developmental values**	3.57	4.21	3.36	3.50	4.07	4.36	4.00	3.64	3.43	4.07
**Coupling Degree C**	0.97	0.87	0.39	0.96	1.00	1.00	0.25	0.91	0.95	0.77
**Coordination Index T**	0.18	0.57	0.13	0.21	0.72	0.99	0.32	0.20	0.12	0.43
**Coupling-Coordination Degree D**	0.42	0.70	0.22	0.45	0.85	0.99	0.28	0.43	0.33	0.58
**Level of Coupling-Coordination**	Near Disarray	Basic Coordination	Moderate Disarray	Near Disarray	Good Coordination	Excellent Coordination	Moderate Disarray	Near Disarray	Mild Disarray	Marginal Coordination

In order to provide a more objective and detailed analysis of the coupling and coordination between these subsystems, the uniform distribution function method is applied. This method reclassifies the derived coupling and coordination degrees into graded judgments for Coupling Degree C and Coordination Index T. These classifications serve as the primary basis for proposing strategies to enhance the construction of the cultural landscape system.

**Fig 8:**
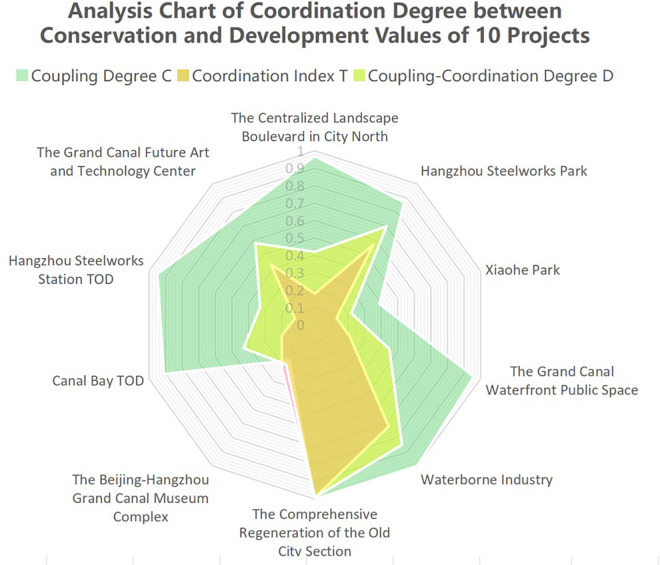
Analysis results of the conservation-development value coupling and coordination for 10 cultural landscape projects of Hangzhou Section.

Based on the evaluation results, Coordination Index T reveals distinct levels among the projects. The Comprehensive Enhancement for the Old City Section and Waterborne Industry exhibit high coordination indices. Meanwhile, Hangzhou Steelworks Park and the Grand Canal Future Art and Technology Center fall in the middle range. The remaining six projects show lower coordination indices. In terms of Coupling Degree C, several projects demonstrate higher levels of conservation and development coupling. These include the Comprehensive Enhancement for the Old City Section, Waterborne Industry, the Grand Canal Waterfront Public Space, the Centralized landscape boulevard in City North, Hangzhou Steelworks Station TOD, Canal Bay TOD, Hangzhou Steelworks Park, and the Grand Canal Future Art and Technology Center. In contrast, Xiaohe Park and the Beijing-Hangzhou Grand Canal Museum Complex (Fig 8). The results of Coupling Degree C and Coordination Index T show three distinct types: higher conservation and development coupling with lower coordination index, higher coupling and coordination index, and lower coupling and coordination index. In the following section, this paper conducts classification analysis and proposes countermeasure suggestions tailored to these three types (Table 8).

**Table 8:** Conservation-development coupling and coordination strategies for 10 cultural landscape projects of the Grand Canal (Hangzhou Section).

Project name	Coupling level	Coordination level	Type of strategy
**The Centralized landscape boulevard in City North**	High	Low	Development-oriented, integrated and optimized
**Hangzhou Steelworks Park**	High	Medium	Development-oriented, integrated and optimized
**Xiaohe Park**	Low	Low	Make up for shortcomings, improve quality
**The Grand Canal Waterfront Public Space**	High	Low	Development-oriented, integrated and optimized
**Waterborne Industry**	High	High	Integrated preservation and utilization, synergistic enhancement
**The Comprehensive regeneration of the Old City Section**	High	High	Integrated preservation and utilization, synergistic enhancement
**The Beijing-Hangzhou Grand Canal Museum Complex**	Low	Low	Make up for shortcomings, improve quality
**Canal Bay TOD**	High	Low	Development-oriented, integrated and optimized
**Hangzhou Steelworks Station TOD**	High	Low	Development-oriented, integrated and optimized
**The Centralized landscape boulevard in City North**	High	Low	Development-oriented, integrated and optimized

### Analysis of cultural landscape strategies based on multi-dimensional values

#### Integration of conservation and utilization, synergistic enhancement.

Projects with a high coupling degree and coordination index exhibit comprehensive value where conservative and developmental aspects synergistically enhance each other. The projects of this kind are well-developed and can be strategically fine-tuned based on spatial development needs and project positioning, achieving a coordinated advancement in integrated conservation and utilization, ushering in a new development paradigm.

The Comprehensive Enhancement for the Old City Section exemplifies this synergy by prioritizing the enhancement of conservative and developmental values. Emphasizing the preservation of natural, historical, cultural, and spiritual values as primary resources, the project aims to bolster landscape and social values while cautiously managing economic development. This approach integrates canal conservation and utilization, establishing it as a pivotal spiritual and cultural asset supporting project development.

Similarly, Waterborne Industry project leverages its historical, cultural, and spiritual values as foundational resources. It focuses on enhancing landscape, economic, and social values to integrate and promote the water industry along the Grand Canal Poetry Road and Qiantang River Poetry Road. This initiative aims to showcase the “Poetry and Painting of Zhejiang” as a prestigious cultural hallmark.

### Development-oriented, integrated and optimized

Projects with a higher degree of conservation and development coupling but a lower coordination index often indicate coupled conservation and development values with room for improved utilization of existing resources and potential for value excavation. For such projects, optimizing developmental value serves as a focal point to comprehensively enhance the coordination and coupling system of cultural landscape conservation and development along the canal.

The Centralized landscape boulevard in City North illustrates this scenario. It faces challenges such as extensive residential buildings with insufficient setback impacting its landscape value, inadequate conservative elements, and a lack of prominent civic imagery. To address these issues, an orientation towards development should prioritize enhancing social values such as public services, social education, and regional synergy. Comprehensive enhancements should aim to bolster both conservative and developmental values, enhancing historical, cultural, and spiritual values through the creation of cultural experience spaces. This approach aims to strengthen the coupling and coordination of the dual conservation and development systems, fostering a modern industrial canal culture boulevard that integrates display, service, and educational functions to promote the development of Hangzhou’s northern urban area.

Hangzhou Steelworks Station TOD project, although yet to commence construction, requires a development-oriented approach to harness the spiritual and cultural values of Hangzhou Steel remains as primary resources. Emphasizing economic and landscape values will be crucial in addressing its current mildly dysfunctional status. Strategies should involve tapping into regional cultural heritage, enhancing public services, optimizing historical, cultural, and social values to promote the project’s sound development and benign growth. As both projects are in the construction phase, this paper serves as a process value assessment influencing their developmental trajectory. The ongoing advancement of these projects will mutually influence their value assessment and construction practices, necessitating ongoing adjustments accordingly.

### Remedying shortcomings, improving quality

Projects with a low coupling and coordination degree often exhibit significant deficiencies in their value systems, resulting in a lower overall value of the dual conservation and development systems and mutual constraints. These projects should prioritize addressing their shortcomings and weaknesses to progressively optimize and enhance their overall quality.

Xiaohe Park project redeveloped Xiaohe petrol depot, a historical building complexes, for modern regeneration. However, it faces challenges such as poor articulation with the surrounding Xiaohezhi Block and other areas, which diminishes its landscape style. To remedy this, the project should focus on enhancing natural, landscape, historical, and cultural values. Addressing the current moderate dislocation of its dual-system approach involves bolstering spiritual, economic, and social values, optimizing site buildings, and improving the surrounding environment. These efforts aim to gradually enhance the overall quality of the project along the canal and promote its healthy development. Situated at the core intersection of river and bay corridors, the Xiaohe Petro Depot historical building complex presents an opportunity to establish a cultural landmark and family park. This initiative integrates functions such as nature preservation, landscape display, cultural experiences, and historical revitalization.

Similarly, the Beijing-Hangzhou Grand Canal Museum Complex, despite its significant volume impacting river and bay view corridors, occupies a site earmarked for new function cultivation. This positioning allows flexibility to appropriately transcend traditional constraints. However, the museum’s construction significantly enhances the cultural landscape value of its site, which stands as a notable achievement. As a pivotal development project along the Grand Canal (Hangzhou section), it should further elevate social and economic values to lead regional development. Addressing the current moderate dissonance between its conservative and developmental systems, the museum should augment historical, cultural, and spiritual values through regional cultural heritage exploration and canal culture displays. Soft landscape interventions should enhance landscape value, fostering coordinated development of both conservation and development systems. This approach aims to establish a landmark project that integrates cultural heritage and development.

## Conclusion

This study has explored the value evaluation framework for the conservation and development of cultural landscapes, focusing on the Hangzhou section of the Grand Canal. Through an analysis of 10 cultural landscape projects, it has investigated strategies for enhancing these landscapes, emphasizing integration of preservation and use, development orientation and comprehensive optimization, and remedying shortcomings to improve quality. Hangzhou section of the Grand Canal, characterized by its interdependence of city and river, is entering a new phase of establishing a national cultural park. Cultural landscape construction and regeneration projects along the canal must prioritize value coordination, further enhancing development while maintaining control. This approach seeks to explore pathways for constructing and developing new functional zones within controlled conditions, aiming to create a canal landscape system that integrates seamlessly with urban life and is accessible to citizens.

In conclusion, this research underscores the importance of harmonizing conservation and development efforts in cultural landscapes. By implementing the strategies identified, stakeholders can effectively navigate the complexities of enhancing cultural heritage while promoting sustainable urban integration along the Grand Canal.
